# Group Factor Analysis for Alzheimer's Disease

**DOI:** 10.1155/2013/428385

**Published:** 2013-03-05

**Authors:** Wei-Chen Cheng, Philip E. Cheng, Michelle Liou

**Affiliations:** Institute of Statistical Science, Academia Sinica, Taipei 11529, Taiwan

## Abstract

For any neuroimaging study in an institute, brain images are normally acquired from healthy controls and patients using a single track of protocol. Traditionally, the factor analysis procedure analyzes image data for healthy controls and patients either together or separately. The former unifies the factor pattern across subjects and the latter deals with measurement errors individually. This paper proposes a group factor analysis model for neuroimaging applications by assigning separate factor patterns to control and patient groups. The clinical diagnosis information is used for categorizing subjects into groups in the analysis procedure. The proposed method allows different groups of subjects to share a common covariance matrix of measurement errors. The empirical results show that the proposed method provides more reasonable factor scores and patterns and is more suitable for medical research based on image data as compared with the conventional factor analysis model.

## 1. Introduction

Modern medical imaging techniques are capable of measuring human brain in vivo [1]. For instance, magnetic resonance (MR) imaging measures nuclei of atoms, and positron emission tomography detects the positron-emitting radionuclides to construct three-dimensional images. The imaging procedures are designed and settled before medical or cognitive experiments. Once the protocol is established, the laboratory and the hospital begin to recruit a variety of subjects of interest into experimental sessions. Errors resulting from individual scans are actually generated from common sources, such as the scanner, protocol, and software. Initial classification of subjects into groups can be realized by using clinical diagnosis, which may be uncertain to some extent, provided by physicians along with subjects' anamnesis.

Conventional factor analysis [2] models reduce high-dimensional data into a few latent variables and assume that data **x** were generated by a set of unobserved independent unit-variance Gaussian source **f** plus uncorrelated zero-mean Gaussian random noise **u**, **x** = *L* **f** + **u**, where *L* is the factor loading matrix. The sample covariance of **x** can be expressed as *LL* ^*T*^ + Ψ, where Ψ is a diagonal covariance matrix of random noises. The goal of factor analysis is to find *L* and Ψ that maximally fit the sample covariance [3–5]. The EM algorithm was proposed to estimate the matrices [6]. Factor analysis is commonly applied to the dataset as a whole or to different groups of data separately, which may result in factor patterns hard to interpret and limit the potential use of the method in a wider range of medical applications. In this study, we propose a mixture factor analysis model (MFAM) to assign a common covariance matrix of noises or measurement errors to different groups of subjects but to allow individual groups having their own latent structures. In the empirical application, we analyzed an Alzheimer's disease (AD) dataset by first extracting the volumetric information from MR anatomical images for both healthy controls and the patients suffering either AD or mild cognitive impairment, followed by applying the proposed MFAM to the volumetric data.

## 2. Material and Method

### 2.1. The Model

Let *M* be the number of subject groups. To find multiple sets of factor loadings, {*L*_*j*_; *j* = 1,…, *M*}, with the **f** scores distributed as Gaussian within each group, the data vector can be decomposed into a linear combination of factor loadings for each group [7, 8], that is, *L*_*j*_ ∈ *R* ^*D*×*K*^,

(1) $$\begin{array}{c} x=\sum_{j}^{}\pi_{j}\left(\mu_{j}+L_{j}\times f\;|\;w_{j}\right)+u, \end{array}$$

where **x** is *D*-dimensional and each factor scores **f** | *w*_*j*_ has *K* variables, that is, **f** ∈ *R* ^*K*^. The parameter ***π*** is associated with the proportion of subjects in the *j*th group, *π*_*j*_ = *p*(*w*_*j*_). The indicator variable *w* is one, *w*_*j*_ = 1, when the data belongs to *j*th group, otherwise *w* is set to zero, *w*_*j*_ = 0. The formula (1) using ***π*** introduces the main difference from previous mixture models of factor analysis. The data vector **x** need not be centered and the mean of the *j*th group data is ***μ***_*j*_. The covariance matrix of residuals **u** is a diagonal matrix Ψ = diag⁡[Ψ_1_, Ψ_2_,…, Ψ_*D*_]. The data distribution can be expressed as

(2) $$\begin{array}{c} P\left(x\right)=\sum_{j=1}^{M}\int P\left(x\;|\;f,w_{j}\right)P\left(f\;|\;w_{j}\right)p\left(w_{j}\right)df. \end{array}$$

In this work, capitalized *P* denotes the probability function of a vector or a matrix and lowercase *p* denotes the probability function of a scalar. The factor scores are assumed to be distributed as Gaussian

(3) $$\begin{array}{c} P\left(f\;|\;w_{j}\right)=N\left(0,I\right),\;\forall j. \end{array}$$

The notation *I* is the identity matrix of order *D*. The distribution of data **x** in each group is given by

(4) $$\begin{array}{c} P\left(x\;|\;f,w_{j}\right)=N\left(\mu_{j}+L_{j}f_{j},\Psi \right). \end{array}$$

Based on the MFAM (2), the likelihood function *Q* is as follows:

(5) Q=E[∏i=1N∏j=1M{(2π)−D/2|Ψ|−1/2exp⁡[−(xi−μj−Ljfi)TΨ−1                    ×(xi−μj−Ljfi)]}wj],

where *E* denotes the expectation. The *N* is the number of data vectors (subjects) with subscript *i* for the *i*th subject. We need to compute the expectation of the variables,

(6) $$\begin{array}{c} E\left(w_{j}f_{i}\;|\;x_{i}\right)=E\left(w_{j}\;|\;x_{i}\right)E\left(f_{i}\;|\;w_{j},x_{i}\right). \end{array}$$

To estimate *Q* in (5), the posterior probability of the *j*th group is calculated as

(7) P(wj ∣ x)=P(x ∣ wj)P(wj)P(x)=πjN(x−μj,LjLjT+Ψ)∑uπuN(x−μu,LuLuT+Ψ),

where the probability of **x** given *w*_*j*_ is

(8) $$\begin{array}{c} P\left(x\;|\;w_{j}\right)=N\left(x-\mu_{j},L_{j}L_{j}^{T}+\Psi \right). \end{array}$$

The parameters ***π*** in (7) is the prior probability derived from the clinical diagnosis. Therefore, the expectation of *w*_*j*_ given **x**_*i*_ in (6) is proportional to the numerator in (7),

(9) $$\begin{array}{c} h_{ij}=E\left[w_{j}\;|\;x_{i}\right]\propto \pi_{j}N\left(x_{i}-\mu_{j},L_{j}L_{j}^{T}+\Psi \right). \end{array}$$

To calculate (6), we consider that the posterior probability of **f** given **x** is

(10) P(f ∣ x)=P(x ∣ f)P(f)P(x)∝exp⁡(−[fT(LTΨ−1L+I)f−2fTLTΨ−1x]).

After some arithmetic calculation, *P*(**f** | **x**) can be expressed as

(11) $$\begin{array}{c} P\left(f\;|\;x\right)\sim N\left(R^{-1}L^{T}\Psi^{-1}x,R\right), \end{array}$$

where *R* = (*L* ^*T*^Ψ^−1^ *L* + *I*). Hence, the expectation of **f** given **x** is

(12) $$\begin{array}{c} E\left[f\;|\;x\right]=R^{-1}L^{T}\Psi^{-1}x. \end{array}$$

From above, *E*  (**f**_*i*_ | *w*_*j*_, **x**_*i*_) in (6) is calculated as

(13) $$\begin{array}{c} E\left(f_{i}\;|\;w_{j},x_{i}\right)=R_{j}^{-1}L_{j}^{T}\Psi^{-1}\left(x_{i}-\mu_{j}\right), \end{array}$$

where *R*_*j*_ = (*L*_*j*_^*T*^Ψ^−1^ *L*_*j*_ + *I*), according to (12).

There is no constraint on those factor loadings *L*_*j*_. The estimation of *L*_*j*_ is simply the maximum of *Q*. A convenient way to express *Q* in (5) is achieved by setting ${\tilde{f}}_{i}={\left[f_{i}^{T}\;1\right]}^{T}$ and ${\tilde{L}}_{j}=\left[L_{j}\;\mu_{j}\right]$. The expected log likelihood function can be expressed as

(14) E[log⁡Q] =E[log⁡⁡∏i=1N∏j=1M{(2π)−D/2|Ψ|−1/2exp⁡[−12(xi−L~jf~i)TΨ−1                         ×(xi−L~jf~i)]}wj] =−D×N2×log⁡(2π)−N2log⁡|Ψ|  −∑i,j12hijxiTΨ−1xi−hijxiTΨ−1L~jE[f~i ∣ xi,wj]  +12hij×trace[L~jTΨ−1L~jE[f~if~iT ∣ xi,wj]].

To maximize *Q* with respect to ${\tilde{L}}_{j}$, we equate the derivative of (14) to zero,

(15) ∂log⁡E[Q]∂L~j=−∑ihijΨ−1xiE[f~i ∣ xi,wj]T +hijΨ−1L~jE[f~if~iT ∣ xi,wj]T=0⇒L~j=(∑ihijxiE[f~i ∣ xi,wj]T)    ×(∑shsjE[f~if~iT ∣ xs,wj]T)−1,

where

(16) $$\begin{array}{c} E\left[{\tilde{f}}_{i}\;|\;w_{j},x_{i}\right]={\left[\begin{array}{cc} E{\left[f_{i}\;|\;w_{j},x_{i}\right]}^{T} & 1 \end{array}\right]}^{T}. \end{array}$$

All the variables are estimated by the EM algorithm. In the E-step, the algorithm computes the expectation of the factor scores in (6) and the second moment of the scores,

(17) $$\begin{array}{c} E\left[w_{j}ff^{T}\;|\;x\right]=E\left[w_{j}\;|\;x\right]E\left[ff^{T}\;|\;w_{j},x\right], \end{array}$$

by

(18) E[wjfifiT ∣ xi]=hijCov(fi ∣ wj,xi)+hijE[fi ∣ wj,xi]E[fi ∣ wj,xi]T.

The covariance matrix of residual, Ψ, can be estimated by its inverse matrix,

(19) ∂Q∂Ψ−1=−N2Ψ−12∑i,jhijxixiT+hijxiE[f~i ∣ xi,wj]TL~jT−12hijL~jE[f~if~iT ∣ xi,wj]TL~jT=0.

Substituting (15) for ${\tilde{L}}_{j}$ and making constraints on the diagonal of Ψ, we obtain

(20) $$\begin{array}{c} \Psi =\frac{1}{N}\mathrm{diag}\left(\sum_{i,j}^{}h_{ij}\left(x_{i}-{\tilde{L}}_{j}E\left[{\tilde{f}}_{i}\;|\;x_{i},w_{j}\right]\right)x_{i}^{T}\right). \end{array}$$

The prior probability *p*(*w*_*j*_) should be proportional to the clinical diagnosis such that the estimation of the factor loadings and the factor scores can capture the latent factors of different disease groups. The proposed model also carries the same indeterminacy problem associated with factor patterns; that is there exist numerous orthogonal transformations to rotate the matrix of factor loadings without changing the maximum of *Q* [9]. Considering *H* be any *K* × *K* orthogonal matrix, *HH* ^*T*^ = *H* ^*T*^ *H* = *I*. Equation (1) can be written

(21) x=∑jπj(μj+Lj×(HHT)×f ∣ wj)+u=∑jπj(μj+Lj∗×f∗ ∣ wj)+u,

where *L*_j_* = *L*_*j*_ *H* and f* | *w*_*j*_ = *H* ^*T*^ × **f** | *w*_*j*_. The assumption, f* | *w*_*j*_ ~ *N*(0, *I*), is kept. The covariance of **x** is *L*_j_*(*L*_j_*)^*T*^ + Ψ = *L*_*j*_ *HH* ^*T*^ *L*_*j*_^*T*^ + Ψ = *L*_*j*_ *L*_*j*_^*T*^ + Ψ, which remains the same. Therefore, there are infinite equivalent solutions to satisfy the maximum of (5). Imposing reasonable constraints to identify a set of model parameters can make the factor loadings scientifically interpretable. A widely used approach for a simple factor structure is realized by setting some factor loadings to hypothetical values such as zeros.

The permutation and changing the sign of columns in the factor loading matrix with factor scores does not affect the model at all and the algorithm will yield the same solution. In order to realize consistent, interpretable, and comparable results, we suggest to recursively test all combinations to find the one of them that has the highest similarity among *M* factor loading matrices so that we can find a coherent interpretation for different groups of subjects. Each pair of factor loading and factor scores can be multiplied by either +1 or −1. The *M* sets of loadings has (2^*K*^)^*M*^ combinations. The possible permutation of the *M* set of loadings is the factorial of *K*. The complexity of the recurrence is therefore 2^*KM*^ × (*K*!)^*M*^. The problem can be formulated as a bipartite matching and the Hungarian algorithm can find the match in a lower complexity.

### 2.2. Data Description

The T1-weighted MR images of 416 subjects were downloaded from the Open Access Series of Imaging Studies [10], which is publically available for analysis. All the T1-weighted images were acquired on a 1.5-T Siemens Vision scanner. Among all 416 subjects, there are 316 normal subjects (average age: 45.09 ± 23.90), 70 subjects who have been clinically diagnosed with very mild AD (average age: 76.21 ± 7.19), and 30 are with moderate AD (average age: 78.03 ± 6.91). The proportions of each type of subject are ***π*** = [75.96%,16.83%,7.21%]^*T*^. Multiple intrasession acquisitions provide extremely high signal-to-noise ratio, making the data amenable to our analysis. The available images were provided skull stripped, gain field corrected, and registered to the atlas space of Talairach and Tournoux [11] with a 12-parameter rigid affine transform. The resolution of the images is 176 × 208 × 176. The number of voxels, which is more than six million, is much larger than the number of subjects. We extracted the clinically and psychologically interested regions instead of processing whole voxels in the image. The subcortical structures are extracted by the segmentation method [12] which uses manually labeled image data as priori information for a Bayesian framework that utilizes the principles of the active shape and appearance models. The size of a subcortical region was calculated by multiplying the voxel size and the number of voxels in the region. Fifteen subcortical regions were successfully extracted.

**Figure 1:**
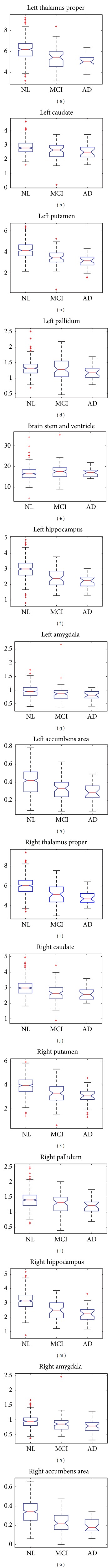
The plots of means and standard deviations for the three groups in different subcortical structures.

According to a demographic study by the National Institute on Aging and Alzheimer's Association based on the data collected in the Chicago Health and Aging Project, the prevalence of dementia among individuals aged 71 and older was 13.9%, and AD (Alzheimers disease) was 9.7% [13]. The study was based on a sample of 856 individuals. The ***π*** was estimated to be [76.4%,13.9%,9.7%]^*T*^ which is close to the statistics in our empirical data. The data vector of each subject had fifteen dimensions, each corresponding to the volume size of a subcortical structure divided by the estimated total intracranial volume. The average size of all of the intracranial volume is 1480.5 cm^3^. The intracranial volume is estimated by the linear registration from a manually measured intracranial volume of a standard brain to the individual brain [14]. The analysis of variance (ANOVA) of the data for each structure were calculated and shown in Table 1 and Figure 1. The smaller *p* value indicates high probability of inequality of the structure size among the three groups.

We subtracted the mean from the data and used the remainder for analysis. Using the covariance matrix of the data to estimate the factor scores would cause that a few structures dominate the factor loadings; therefore, we divided each dimension by its standard deviation to compel each of them to have unit variance. After the algorithm converged, we used varimax rotation [15], which transforms the loadings into the space that maximizes the variance, to rotate the factor loadings. Given data, the expectation of its type was set to

(22) [hi1,hi2,hi3]={[1,0,0]if  subject  i  belongs  to  NL [0,1,0]  if  subject  i  belongs  to  vAD [0,0,1]if  subject  i  belongs  to  AD.

## 3. Results

**Figure 2:**
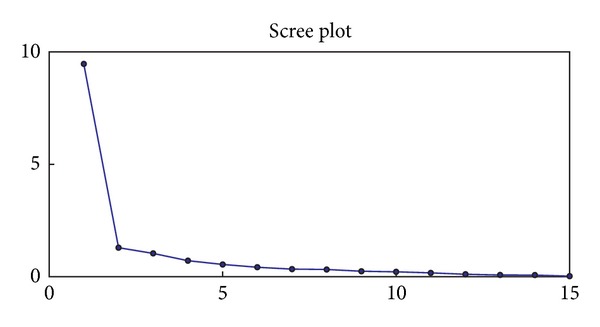
The scree plot for the ordered eigenvalues.

**Figure 3:**
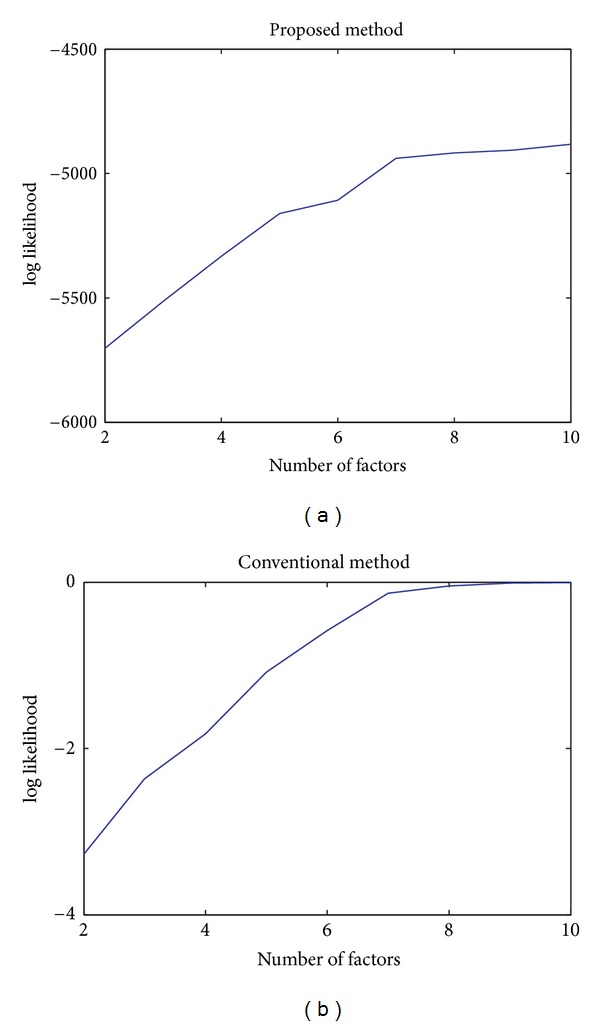
The two curves record the approximate value of log likelihood for two methods.

Figure 3 shows the trend of the likelihood climbs as adding the number of factors in the analysis. In the scree plot in Figure 2, three eigenvalues of the covariance matrix of the whole dataset are greater than one and the cumulative percentage of variance from the largest three eigenvalues reaches 78%. Thus we set *K* = 3 in this analysis.

**Figure 4:**
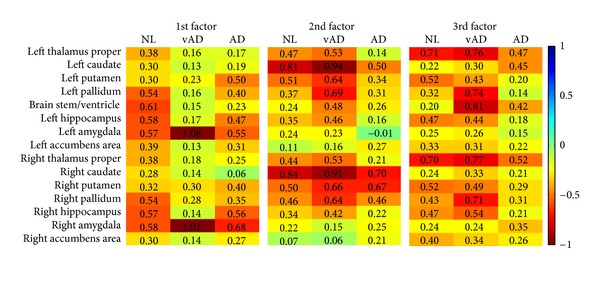
The factor loading matrices for the three groups.

The factor loadings for the three groups are shown in Figure 4, in which the vertical axis marks the fifteen regions. The vAD denotes the group of very mild AD. The log likelihood in (5) after the algorithm converges is −5475.814. The loading of structures has symmetric property and usually the right and left structures have similar loadings. Using the factor loadings to estimate ***π*** and the expected group information given **x** by (9), we obtain the adjusted and turned proportions as [82.89%,7.97%,9.14%]^*T*^. This may suggest the underlying variation among different groups of subject and need further investigation. Note that the reestimated proportion *h*_*ij*_ is not binary anymore.

**Figure 5:**
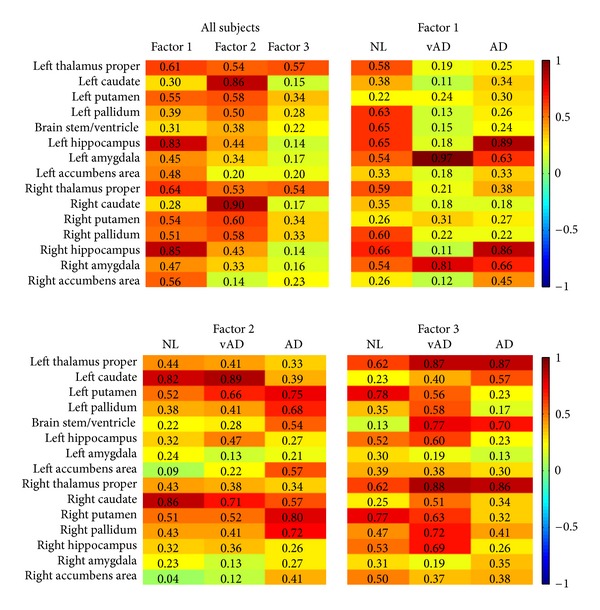
The result of maximum likelihood method and rotated by varimax with Kaiser normalization. The results of healthy controls are not unique and unstable.

We show the results of conventional factor analysis in Figure 5 as a comparison. The program run on the mild AD patients in the dataset cannot achieve reproducible results; therefore, the quantity of mild AD's results in Figure 5 varies from time to time. The analysis for the AD group cannot converge, however the factor loadings are reproducible. The distance of whole factor loading matrices among the three groups for conventional factor analysis is 5.28 while the proposed method is 4.54. The correlation of the three-factor loading matrix estimated by conventional factor analysis methods is [*C*_12_, *C*_13_, *C*_23_] = [0.3879,0.1698,0.3633]. The correlation by proposed method is [0.5388,0.5564,0.4986].  Table 2 lists the *p* values for all factors by the Kolmogorov-Smirnov test [16] on the factor scores against a Gaussian distribution. The test examines the difference between input distributions and a Gaussian distribution. The smaller the *p* values, the more strongly the test rejects the Gaussian assumption. The algorithm tries to search for loads with normally distributed factor score, hence large *p* indicates the factor fit well to Gaussian distribution.

**Figure 6:**
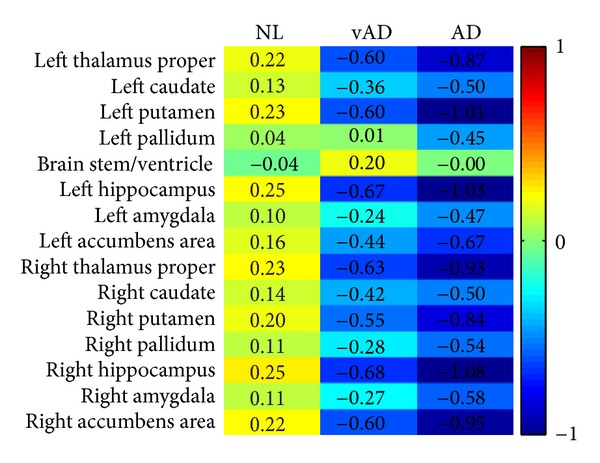
The cluster means of the three groups.

**Figure 7:**
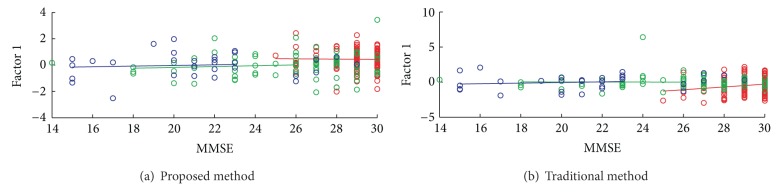
Correlations of factor scores with the MMSE scores. The red color denotes healthy subjects; the green color denotes very mild AD patients; the blue color denotes the moderate AD patients. The regression lines of the three groups by the (a) proposed method and (b) conventional method are also plotted in the figures.

The means (centers) of the clusters are shown in Figure 6. The means are near the origin and include negative value because the data are standardized by the subtraction of the overall mean of the data in the preprocess stage. The yellow color in the first column indicates that healthy controls have larger sizes in subcortical structures, and the second and the third columns indicate that the patients have smaller sizes in different subcortical regions in general. The AD patient has very small thalamus, putamen, and hippocampus. The hippocampus is related to memory and learning. The putamen is a structure involved in the regulation of voluntary movement. The abnormal pallidum in Figure 4 can cause movement disorders. Figure 7 shows the associations of first factor scores with the score of minimental state examination (MMSE) by both methods.

## 4. Conclusions

The proposed method finds closer and more correlated factor loadings than the conventional method because it considers the same error matrix for different groups of data. The result of conventional factor analysis having higher normality for AD patients than normal subjects is less convincing. Conventional factor analysis that decomposes the observed data together intermixes the latent factors. Taking the data apart will misseparate the noise. This work proposed using a mixture model of factor analysis method for neurodegenerative disease research by showing highly correlated factor loading across different groups of subjects and together with proper normality of the factor scores.

## Figures and Tables

**Table 1:** The ANOVA results for the three groups in different subcortical structures.

Structure	*p*-value
Left thalamus proper	2.0183 × 10^−15^
Left caudate	1.3736 × 10^−5^
Left putamen	6.9145 × 10^−18^
Left pallidum	0.0351
Brain stem and ventricle	0.1707
Left hippocampus	1.5656 × 10^−20^
Left amygdala	9.1999 × 10^−4^
Left accumbens area	1.0958 × 10^−8^
Right thalamus proper	2.6864 × 10^−17^
Right caudate	1.6556 × 10^−6^
Right putamen	1.6447 × 10^−13^
Right pallidum	8.8771 × 10^−5^
Right hippocampus	3.5498 × 10^−22^
Right amygdala	6.5971 × 10^−5^
Right accumbens area	8.7323 × 10^−17^

**Table 2:** The normality test of factor score by Kolmogorov-Smirnov test.

*p* value	Proposed method			Traditional method		
	Factor 1	Factor 2	Factor 3	Factor 1	Factor 2	Factor 3
NL	0.20	0.12	0.53	0.51	0.19	0.82
vAD	0.00	0.55	0.29	0.54	0.44	0.30
AD	0.02	0.14	0.14	0.81	0.89	0.83
